# Supplementary material for: Uniformly Dispersed Sb-Nanodot Constructed by In Situ Confined Polymerization of Ionic Liquids for High-Performance Potassium-Ion Batteries
Source: Molecules. 2023 Jul 5;28(13):5212. doi: 10.3390/molecules28135212 (PMC10343779; doi:10.3390/molecules28135212)
Supplement: Supplementary file 1 [file molecules-28-05212-s001.zip › molecules-2447327-supplementary.pdf]

Electronic Supplementary Information

Cunliang Zhang [1], Zhengyuan Chen [2], Haojie Zhang [1], Yanmei Liu [3], Wei Wei [1], Yanli Zhou [1,*] and Maotian Xu [1,*]

[1] School of Chemistry and Chemical Engineering, Henan Key Laboratory of Bimolecular Reorganization and Sensing, Henan Engineering Center of New Energy Battery Materials, Shangqiu Normal University, Shangqiu 476000, People's Republic of China
[2] School of Petrochemical Engineering, Liaoning Petrochemical University, Fushun 113001, People's Republic of China
[3] Department of Public Science, Shangqiu Medical College, Shangqiu, Henan 476000, People's Republic of China
* Correspondence: Correspondence: zhouyanli@mails.ucas.ac.cn (Y.Z.); umaotian@sqnu.edu.cn (M.X.)

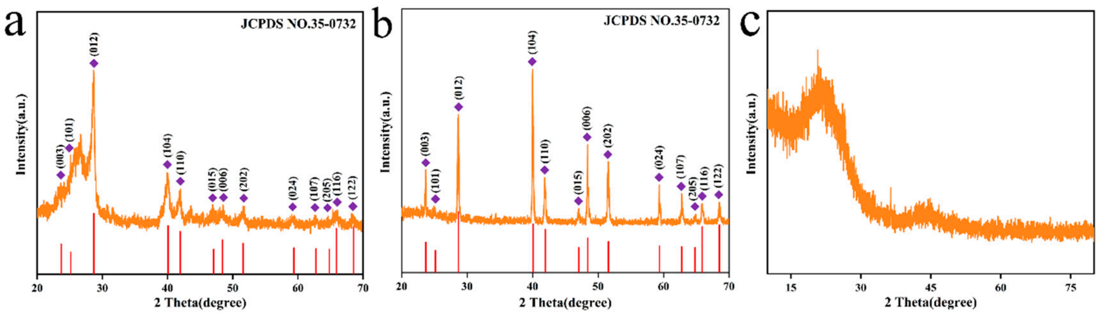

Figure S1 XRD patterns of Sb-RGO (a), Sb (b) and C (c)

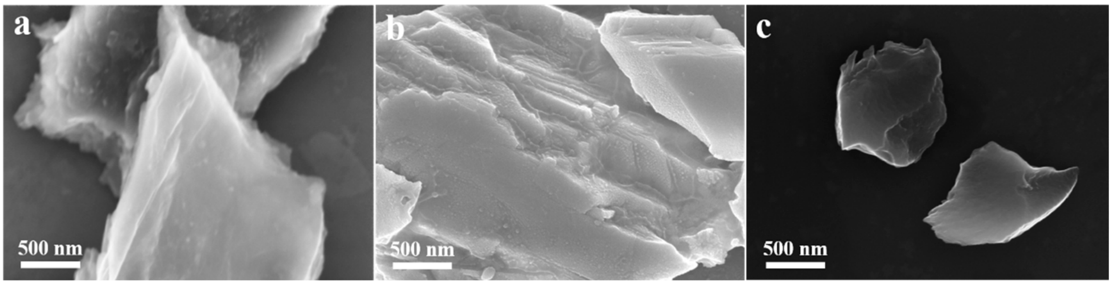

Figure S2 SEM images of Sb-RGO (a), Sb (b) and C (c)

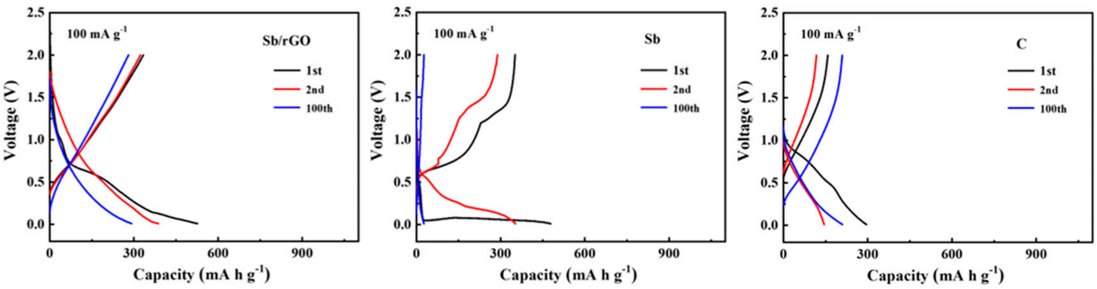

Figure S3 the galvanostatic discharge–charge profiles for the 1st, 2nd and 100th cycles cycles of Sb/rGO, Sb and C electrodes at 100 mA g^{-1}

Table S1 Comparison of cycling performance between the Sb-ND@C and other reported Sb-based electrodes

Material	Preparation method	Sb content in active materials(%)	The proportion of active materials, conductive additive and binder	Cycling performance (mA h g^{-1}) (Based on the whole electrode)	Refs
NP-Sb	vacuum-distillation	100%	6:2:2	190.8/0.1A g^{-1}/50th	S1
Sb-MOFs-NPs@PC	co-precipitation and high temperature annealing	55.8%	8:1:1	221.8/0.1 A g^{-1}/100th	S2
Sb-C-rGO	Ultrasonic spray pyrolysis	50.7%	7:2:1	137.7/0.2A g^{-1}/50th	S3
Sb/C	high-energy ball-milling	50.0%	8:1:1	114.0/0.1A g^{-1}/50th	S4
Sb@C-3DP	Template method	90.0%	8:1:1	366.5/0.05A g^{-1}/50th	S5
Sb@PC	In situ substitution	66.7%	8:1:1	308.4/0.1A g^{-1}/50th	S6
Sb-Nanodot	In Situ Confined	58.7%	8:1:1	486.7/0.5A g^{-1}/50th	This work
